# The authors’ replies to comments on “prenatal exposure to green space and mental health in early adolescence: findings from the TRAILS study”

**DOI:** 10.1093/aje/kwaf219

**Published:** 2025-10-06

**Authors:** Yi Zeng, Gonneke W J M Stevens, Tomáš Paus, Marco Helbich

**Affiliations:** Department of Human Geography and Spatial Planning, Faculty of Geosciences, Utrecht University, Princetonlaan 8a, 3584 CB Utrecht, The Netherlands; Department of Interdisciplinary Social Science, Faculty of Social and Behavioural Sciences, Utrecht University, Padualaan 14, 3584 CH Utrecht, The Netherlands; Centre Hospitalier Universitaire Sainte-Justine, University of Montreal, H3T 1C5 Montreal, QC, Canada; Departments of Psychiatry and Neuroscience, Faculty of Medicine, University of Montreal, H3C 3J7 Montreal, QC, Canada; ECOGENE-21, G7H 7K9 Chicoutimi, QC, Canada; Department of Human Geography and Spatial Planning, Faculty of Geosciences, Utrecht University, Princetonlaan 8a, 3584 CB Utrecht, The Netherlands

We appreciate the thoughtful comments^1^ and critical reflections on our article.^2^ We welcome the opportunity to respond to these points.

We agree that assessing green space at the postal code level has limitations. The use of postal codes was mainly due to the unavailability of precise residential addresses for mothers during their pregnancy. As we noted in the limitations, we were also unable to account for residential mobility during pregnancy. This is also largely ignored by earlier relevant studies,^3^^-^^5^ possibly due to the unavailability of such highly detailed mobility data (eg, relocating dates and locations). While ignoring residential mobility during pregnancy may introduce exposure misclassification, prior studies have found that such misclassification tends to be minor.^6^ Future studies using more granular data on residential addresses and history are needed to replicate our findings.

The commentary indicated the potential overadjustment issue when mutually adjusting for exposure to green space during pregnancy and early adolescence. While the correlation between the two measures is moderately strong (*r* = 0.67), it does not pose a severe multicollinearity issue. Exposure at each time point retained sufficient unique variance after accounting for the shared variance. We carefully built the models stepwise and kept an eye on the precision (ie, standard error) of the result estimates. The standard error for prenatal exposure to green space did not substantially increase after mutual adjustment (0.04 to 0.05). This is further supported by the low values of the variance inflation factor (<4) for both green space measures. In fact, there is always a tradeoff between confounding bias and imprecision.^7^ The more pronounced associations after adjustment may reflect a suppression effect due to the opposite directions of associations at the two time points. Due to the highly similar point estimates for both green space measures, we speculated that both associations are consequences of changes in green space exposure in the intervening years between birth and early adolescence. Interpreting such result patterns as reflecting associations with changes in exposure has also been suggested in previous studies.^8^^,^^9^ To aid interpretation, we conducted a post hoc analysis, reframing the model to express green space change between the two time points. As noted in our article, this was a simple algebraic transformation of the original model rather than a new estimation strategy. Our goal was to clarify the interpretation of the mutual adjustment model by presenting the same relationship in a more intuitive manner. As we already stressed in the article, this result should be interpreted cautiously, and we presented it mainly as a possible explanation for our unexpected finding.

We agree that gestational age and birthweight showed limited variability in our sample, which may explain our null mediation finding. We have also highlighted this possibility in our article. The commentary also noted our decision not to examine threshold effects (eg, for clinically relevant externalizing problems) and interactions between green space with factors in other contexts (eg, schools). This decision was made purposely to avoid an overly complex model because multiple outcomes and mediators were already considered in the models. It is also not uncommon to use continuous or count scores for externalizing problems, as seen in previous TRAILS studies and other relevant research, including one directly comparable to ours, which similarly found that more prenatal exposure to green space was associated with more externalizing problems at ages 6 to 11.^5^

We agree that our adjustment for environmental confounding may be limited. As discussed in our article, urbanicity is a crude proxy for multiple urban-related factors. As such, residual confounding by unmeasured urban exposures may have contributed to our findings. We already emphasized this limitation in the article and called for future research using more spatially resolved environmental exposure data.

Lastly, the prevalence of tobacco and alcohol use is typically low at the age of outcome measurement (age 11). It is typically an age when a few adolescents start to experiment with tobacco and alcohol use; cases with heavy use are typically rare. It is therefore difficult to assess robust dose–response gradients for these outcomes at this young age. Continuous or ordinal measures may be more informative at later ages when substance use is more prevalent.

We are grateful for the constructive feedback from the commentary. We hope this response helps clarify our methodological decisions, the limitations we faced, and highlights both the challenges and opportunities for future research.
